# Accuracy of axial length, keratometry, and refractive measurement with Myopia Master in children with ametropia

**DOI:** 10.1186/s12886-022-02672-9

**Published:** 2022-12-03

**Authors:** Yuhao Ye, Yu Zhao, Tian Han, Xiaoyu Zhang, Huamao Miao, Bing Qin, Xingtao Zhou

**Affiliations:** 1 Department of Ophthalmology and Optometry, Eye and ENT Hospital of Fudan University, Shanghai, China; 2 NHC Key Laboratory of Myopia (Fudan University), Laboratory of Myopia, Chinese Academy of Medical Sciences, 83 Fenyang Road, Shanghai, Shanghai 200031 China; 3 Shanghai Research Center of Ophthalmology and Optometry, Shanghai, China; 4 Shanghai Engineering Research Center of Laser and Autostereoscopic 3D for Vision Care (20DZ2255000), Shanghai, China

**Keywords:** Myopia Master, Axial length, Keratometry, Ametropia

## Abstract

**Purpose:**

To evaluate the accuracy of axial length, keratometry, and refractive measurement with Myopia Master in ametropic children.

**Methods:**

In this randomized prospective cross-sectional study, 125 children with ametropia (250 eyes) were recruited (55 boys and 70 girls; age range: 3–15 years). All examinations were performed under full cycloplegic conditions. Measurements of axial length (AL), keratometry, and autorefraction acquired with the Myopia Master were compared with those from the IOLMaster 500, IOLMaster 700, Nidek ARK-1, and manifest refraction. The differences between the different methods were analyzed, and their correlation was assessed by interclass correlation coefficients (ICCs), Bland–Altman plot, and correlation test.

**Results:**

The ALs (mm) measured with Myopia Master, IOLMaster 500, and IOLMaster 700 were 23.67 ± 1.26, 23.68 ± 1.26, and 23.70 ± 1.25, respectively. The mean values and standard deviations for AL and keratometry readings from these devices were similar (*P* ≥ 0.059). The ICC analysis also revealed high consistency between the measurements (ICC ≥ 0.943). Additionally, the correlation coefficients were relatively high (*r* > 0.9, *p* < 0.001). Although the results of refraction obtained with the Myopia Master were slightly higher than those with manifest refraction (*P* ≤ 0.024), the agreement between these two measurements was excellent (ICC ≥ 0.858). The percentage of points outside the limits of agreements was < 5.22% in Bland–Altman plots for all analyses.

**Conclusions:**

Myopia Master could be a highly efficient tool for clinical use as a three-in-one system (AL, keratometry, and refractive measurements) for screening in children with ametropia.

## Introduction

Myopia has reached epidemic levels worldwide in recent years, and its prevalence is continuing to increase rapidly. A previous study shows that more than 12.8 million adolescents aged 5 to 15 years have myopia worldwide, with the highest prevalence in Southeast and East Asia [1]. The early onset of myopia increases the possibility of developing high myopia in adult life, possibly leading to cataract, glaucoma, retinal detachment, and ultimately, severe visual impairment.

Axial length (AL), one of the most important ocular parameters associated with myopia [2], increases during childhood and adolescence and tends to be stable in adults. The corneal radius of curvature (CR) and spherical equivalent (SE) are two additional indispensable parameters for evaluating myopia. Besides, axial length and the corneal radius of curvature are crucial either for the estimation of the refraction status or myopia progression, because these two parameters are relatively objective and can work as a comparison to minimize subjective error especially in incoordinate subjects. Children, unlike adults, cooperate inadequately with ocular measurements; therefore, they need to be examined with more efficient and accurate equipment. In the past few years, acquiring these ocular parameters with simplicity and efficiency in children has been a continuous focusing point. Additionally, the ability to identify children at high risk of myopia in the early years enables the application of preventative treatments.

Myopia Master (Oculus Optikgeräte GmbH, Wetzlar, Germany) is a relatively new ocular measurement platform that combines AL, CR, and auto-refraction. Myopia Master also provides the estimated SE percentile curve based on ethnicity, age, sex, AL, auto-refraction, and other essential information. However, to the best of our knowledge, no previous studies investigated the accuracy of Myopia Master in acquiring these ocular parameters in children.

Recently, some optical biometry instruments (e.g., IOLMaster 500, IOLMaster 700 [both Carl Zeiss AG, Oberkochen, Germany]) have been widely used to measure AL and CR [3]. Nidek ARK-1 (Nidek Co., Aichi, Japan) is an autorefractor/keratometer platform that combines autorefraction and keratometry [4]. Previous studies demonstrated that all these instruments have high accuracy in measurements, and are valuable tools in clinical applications.

The current study aimed to determine the accuracy of AL, CR, and SE measurements using Myopia Master and assessing the agreement between Myopia Master measurements and those of IOLMaster 500, IOLMaster 700, and Nidek ARK-1.

## Patients and methods

### Patients

The study protocol was approved by the Ethics Committee of Fudan University Eye (2,020,022) and the ENT Hospital Review Board and followed the tenets of the Declaration of Helsinki. Written informed consent was obtained from parents prior to participation in the study.

In this randomized cross-sectional study, 125 patients (55 boys and 70 girls, age range: 3–15 years, 250 eyes) were enrolled at the Eye & ENT Hospital of Fudan University, China, in April 2021. The inclusion criteria were (1) children between 3 and 15 years of age with ametropia including myopia, hyperopia or astigmatism with any degree (> 0.25 D or < -0.25 D) and (2) no history of contact lens use. The exclusion criteria were as follows: (1) previous corneal or intraocular surgery, (2) inflammation of the eye or other ocular diseases, (3) systemic diseases, and (4) contraindications for cycloplegic examination.

### Measurements

For cycloplegia, five eye drops of 1% tropicamide in total were administered to each eye, at 0, 5, 10, 15, and 20 min. Pupil dilation and light reflex were verified 20 min after the last drop. Full cycloplegia was assumed if the light reflex was absent. Two well-trained ophthalmologists and one senior optometrist performed all examinations.

The Myopia Master provides integrated measurements of AL, CR, autorefraction, and estimated SE percentile curve, estimating the risk of myopia in adulthood. It was operated by one ophthalmologist for all patients; the subject was instructed to place their chin on the chin rest and fixate on the target light. The device could measure the AL six times, the CR three times, and autorefraction once in a single session. All results were displayed on the same interface and could be extracted directly.

Another ophthalmologist, blinded to the previous results, measured the ocular parameters using the IOLMaster 500, IOLMaster 700, and Nidek ARK-1. The AL was obtained using the IOLMaster 500 and IOLMaster 700 and the CR using IOLMaster 700 and Nidek ARK-1. The autorefractive error was measured using Nidek ARK-1. Finally, one optometrist measured the manifest refraction using a phoropter (RT-5100, Nidek).

For each device, six measurements with a quality control assessment ≥ 7/9 were accepted per eye and then averaged to obtain the final result.

### Statistical analysis

All statistical analyses were performed using the SPSS software (version 25.0; IBM, Armonk, NY, USA). Descriptive results are presented as mean and standard deviation (SD). The Shapiro–Wilk normality test and the test for homogeneity of variances were performed for all data. Normally distributed data were compared using the paired *t-*test; non-normally distributed data with the Wilcoxon test. The correlations between datasets were calculated with the interclass correlation coefficients (ICCs); a value < 0.4 indicates low consistency, between 0.4 and 0.7 moderate, > 0.7 high consistency [5]. The agreement between devices was evaluated using the Bland–Altman method, with 95% limits of agreement (LoA) referring to the mean ± 1.96 SD. The Pearson’s correlation test was used to investigate the association between variables. Statistical significance was set at *P* < 0.05.

## Results

All patients completed the examinations successfully, and the total data loss for all types was < 5%. Table 1 presents an overview of optical and biometric parameters in two different groups according to gender.

**Table 1:** Axial length (AL), Corneal radius of curvature (CR) and Spherical Equivalent (SE) By Myopia Master in two different groups according to different genders

	Male	Female	*P*-value
Age	8.27±2.68 (3,15)	8.31±2.20 (3,12)	0.95
AL (mm)	24.02±1.20 (21.35,26.86)	23.39±1.23 (20.50,26.74)	<0.001
CR (mm)	7.85±0.23 (7.25,8.64)	7.73±0.24 (7.25,8.62)	<0.001
AL/CR	3.06±0.14 (2.66,3.40)	3.03±0.15 (2.56,3.38)	0.07
SE (D)	-1.45±1.79 (-6.00,5.13)	-1.27±2.35 (-8.63,6.75)	0.50

### Axial length

**Fig. 1:**
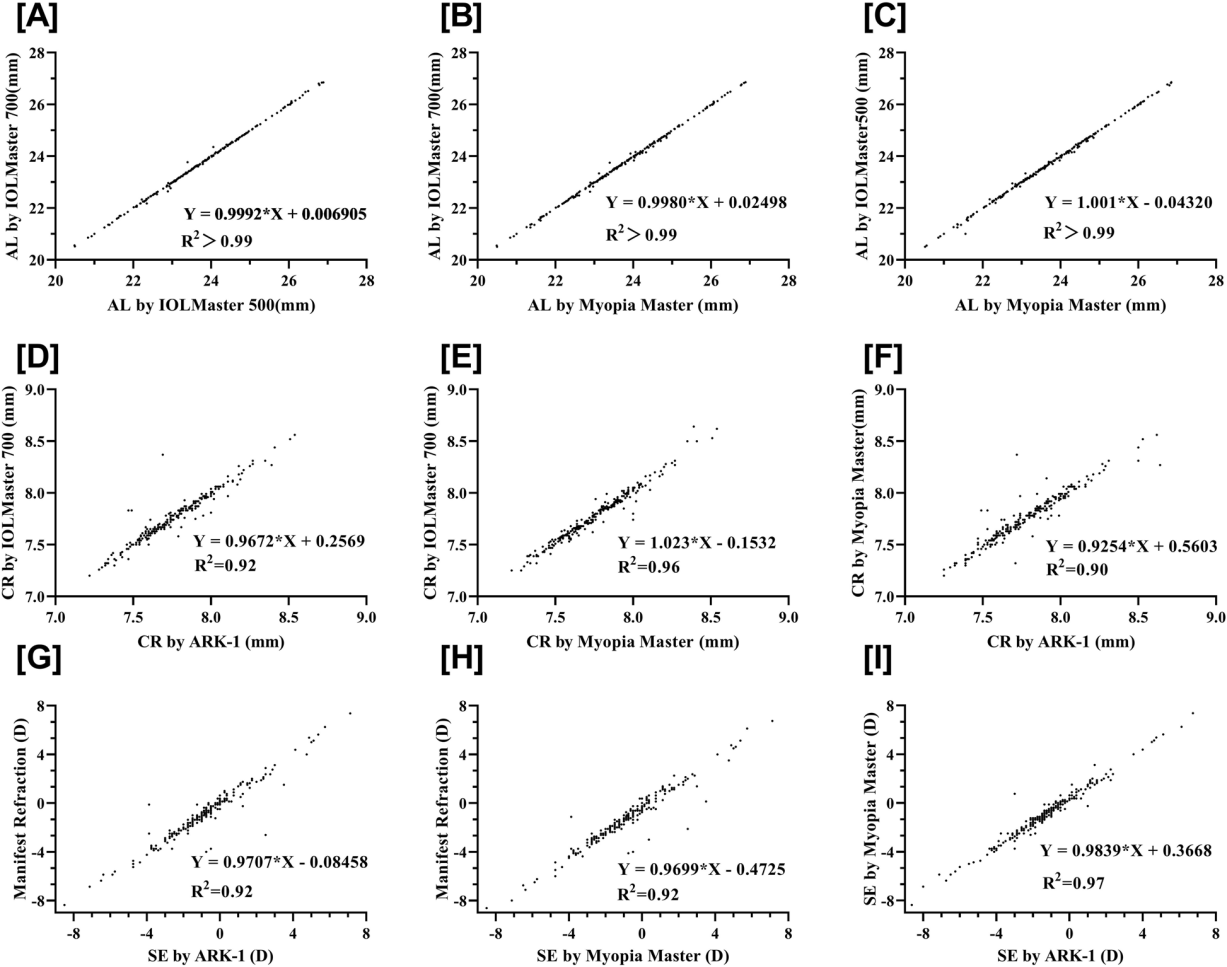
Linear regression analysis of axial length, corneal radius of curvature and spherical equivalent between Myopia Master, IOLMaster 700, IOLMaster 500, Nidek ARK-1, and manifest refraction **A**-**C **Linear regression of AL between IOLMaster 700, IOLMaster 500, and Myopia Master; **D**-**F** Linear regression of CR between IOLMaster 700, ARK-1, and Myopia Master; **G**-**I** Linear regression of SE between IOLMaster 700, IOLMaster 500, and Myopia Master. AL: Axial Length; CR: Corneal radius of curvature; SE: Spherical Equivalent; ARK: Auto Ref/Keratometer.

**Fig. 2:**
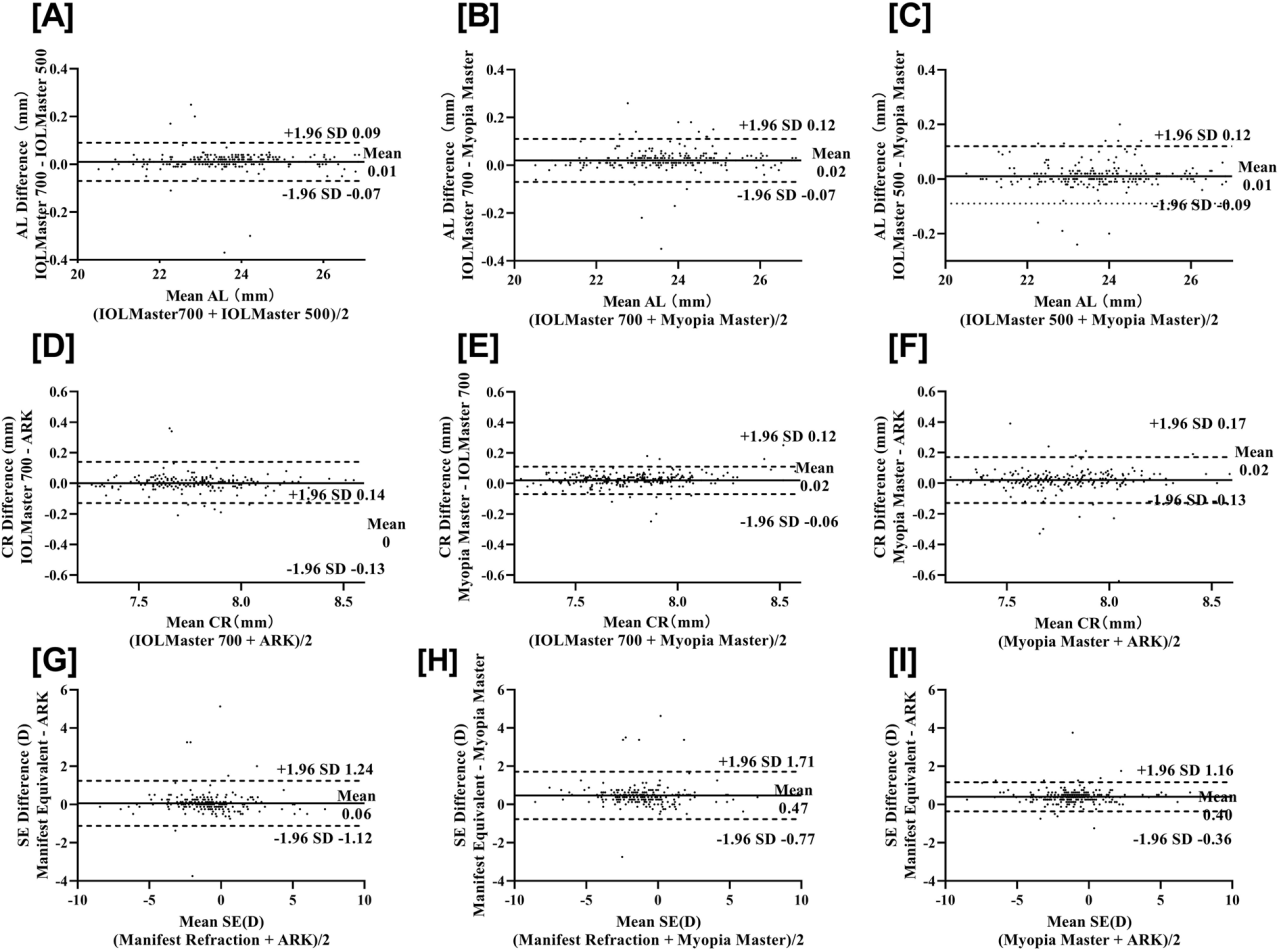
Bland–Altman plots between the measurements acquired by Myopia Master (MM) and the other instruments; the upper and lower dashed lines represent the 95% limits of agreement **A**-**C** Bland-Altman plots of AL between IOLMaster 700, IOLMaster 500, and Myopia Master; **D**-**F** Bland-Altman plots of CR between IOLMaster 700, ARK-1, and Myopia Master; **G**-**I** Bland-Altman plots of SE between ARK-1, manifest refraction, and Myopia Master. AL: Axial Length; CR: Corneal radius of curvature; SE: Spherical Equivalent; ARK: Auto Ref/Keratometer.

Table 2 shows the AL results obtained with the Myopia Master, IOLMaster 700, and IOLMaster 500 with high correlation coefficients between them (all *r* > 0.999, *p* < 0.001; Fig. 1A-C). Significantly longer AL were found in male patients than in female patients (24.02 ± 1.20 mm vs. 23.39 ± 1.23 mm, *P* < 0.001; Table 1). The mean differences between AL readings with these devices were 0.01 (IOLMaster 700 and IOLMaster 500), 0.02 (IOLMaster 700 and Myopia Master), and 0.01 mm (IOLMaster 500 and Myopia Master), respectively. The corresponding LoA were (− 0.07, 0.09), (− 0.07, 0.12), and (− 0.09, 0.12), respectively. The percentages of points outside the LoA were 2.41%, 5.22%, and 4% in Bland–Altman plots (Fig. 2A-C). The ICC analysis showed high consistency between the measurements (Table 2).

**Table 2:** The comparison of axial lengths (AL) between Myopia master, IOLMaster 700 and IOLMaster 500

Device	Mean±SD (min,max)	Compared test (p value)	Correlation test		ICC	Difference of the means	95% LOA	
			r	P			Lower	Upper
Myopia master	23.67±1.26(20.50,26.86)	0.741	all 0.999	all <0.001	0.999 (0.998,0.999)	0.02	-0.07	0.12
IOL Master 700	23.70±1.25(20.49,26.89)							
Myopia master	as above	0.908			0.999 (0.999,0.999)	0.01	-0.09	0.12
IOL Master 500	23.68±1.26(20.52,26.85)							
IOL Master 700	as above	0.819			0.999 (0.999,1)	0.01	-0.07	0.09
IOL Master 500	as above							

*SD* standard deviation, *ICC (A, 1)* intraclass correlation coefficiens (inter-rater reliability, two-way random effect model), *LOA* limit of agreements

### Corneal radius of curvature

The detailed information of the CR results obtained with the Myopia Master, IOLMaster 700, and ARK-1 are displayed in Table 3 with high correlation coefficients (all *r* > 0.946, *P* < 0.001; Fig. 1D-F). Significantly flatter CR values were found in male patients than in female patients (7.85 ± 0.23 mm vs. 7.73 ± 0.24 mm, *P* < 0.001; Table 1). The mean differences between CR readings from these devices were 0 (IOLMaster 700 and ARK-1), 0.02 (IOLMaster 700 and Myopia Master), and 0.02 mm (ARK-1 and Myopia Master), respectively. The corresponding LoA were (− 0.13, 0.14), (− 0.06, 0.12), and (− 0.13, 0.17), respectively. The percentages of points outside the LoA were 3.84%, 4.64%, and 3.30% in Bland–Altman plots (Fig. 2D-F). The ICC analysis showed high consistency between the measurements (Table 3).

**Table 3:** The comparison of corneal radius of curvatures (CR) between Myopia master, IOLMaster 700 and ARK-1

	Device	Mean±SD (min,max)	Compared test (p value)	Correlation test		ICC	Difference of the means	95% LOA	
				r	P			Lower	Upper
K-flat (mm)	Myopia master	7.89±0.26(7.39,8.88)	0.944	0.988	all <0.001	0.994 (0.992,0.995)	0	-0.07	0.08
	IOLMaster 700	7.88±0.26(7.28, 8.88)							
	Myopia master	as above	0.986	0.957		0.978 (0.972,0.983)	0	-0.15	0.15
	ARK-1	7.89±0.26(7.38,8.88)							
	IOLMaster 700	as above	0.912	0.953		0.976 (0.969,0.981)	0.07	-0.15	0.16
	ARK-1	as above							
K-steep (mm)	Myopia master	7.68±0.25(7.11, 8.54)	0.059	0.951		0.974 (0.966,0.980)	0.04	-0.11	0.19
	IOLMaster 700	7.64±0.23(7.09, 8.24)							
	Myopia master	as above	0.136	0.901		0.947 (0.932,0.959)	0.04	-0.17	0.27
	ARK-1	7.64±0.24(7.03,8.28)							
	IOLMaster 700	as above	0.691	0.919		0.958 (0.945,0.967)	0	-0.19	0.19
	ARK-1	as above							
K-mean (mm)	Myopia master	7.78±0.24(7.25, 8.64)	0.364	0.982		0.977 (0.958,0.986)	0.02	-0.06	0.12
	IOLMaster 700	7.76±0.24(7.22, 8.54)							
	Myopia master	as above	0.494	0.946		0.943 (0.923,0.957)	0.02	-0.13	0.17
	ARK-1	7.76±0.24(7.20,8.56)							
	IOLMaster 700	as above	0.819	0.959		0.959 (0.947,0.968)	0	-0.13	0.14
	ARK-1	as above							

*SD* standard deviation, *ICC (A, 1)* intraclass correlation coefficiens (inter-rater reliability, two-way random effect model), *LOA* limit of agreements

### Spherical equivalent

Table 4 shows the SE results obtained with the Myopia Master, manifest refraction, and ARK-1 with high correlation coefficients (all *r* > 0.920, *p* < 0.001; Fig. 1G-I). No significantly different SE values were found between male and female patients (− 1.45 ± 1.79 D vs. − 1.27 ± 2.35 D, *P* = 0.50; Table 1). The mean differences between CR readings with these devices were 0.06 (manifest refraction and ARK-1), 0.47 (manifest refraction and Myopia Master), and 0.40 (ARK-1 and Myopia Master), respectively. The corresponding LoA were (− 1.12, 1.24), (− 0.77, 1.71), and (− 0.36, 1.16), respectively. The percentages of points outside the LoA were 2.87%, 2.46%, and 4.13% in Bland–Altman plots (Fig. 2G-I). The ICC analysis showed high consistency between the measurements (Table 4).

**Table 4:** The comparison of refraction spherical, refraction cylinder and refraction equivalent between Myopia master, ARK-1 and manifest refraction

	Device	Mean±SD (min,max)	Compared test (p value)	Correlation test		ICC	Difference of the means	95% LOA	
				r	P			Lower	Upper
Spherical Refraction	Myopia master	-0.95±2.13(-8.00,7.75)	0.01	0.954	all <0.001	0.976 (0.970,0.982)	0.36	-0.91	1.63
	Manifest refraction	-0.57±2.16(-7.75,8.00)							
	Myopia master	as above	0.016	0.988		0.994 (0.992,0.995)	0.37	-0.29	1.04
	ARK-1	-0.57±2.16(-7.50,8.50)							
	Manifest refraction	as above	0.901	0.953		0.976 (0.969,0.981)	-0.01	-1.30	1.28
	ARK-1	as above							
Cylindrical Refraction	Myopia master	-0.79±0.71(-4.50,0)	**0.002**	0.748		0.858 (0.817,0.890)	0.17	-0.76	1.11
	Manifest refraction	-0.6±0.63(-3.50,0)							
	Myopia master	as above	0.375	0.852		0.933 (0.914,0.948)	0.05	-0.63	0.73
	ARK-1	-0.73±0.68(-4.00,0)							
	Manifest refraction	as above	**0.024**	0.812		0.908 (0.881,0.928)	0.13	-0.61	0.86
	ARK-1	as above							
Spherical Equivalent	Myopia master	-1.35±2.12(-8.63,6.75)	**0.001**	0.960		0.939 (0.799,0.972)	0.47	-0.77	1.71
	Manifest refraction	-0.86±2.11(-8.50,7.13)							
	Myopia master	as above	**0.008**	0.984		0.968 (0.768,0.989)	0.40	-0.36	1.16
	ARK-1	-0.93±2.13(-8.38,7.38)							
	Manifest refraction	as above	0.646	0.960		0.959 (0.947,0.968)	0.06	-1.12	1.24
	ARK-1	as above							

Values with statistical significance are shown in bold

*SD* standard deviation, *ICC (A, 1)* intraclass correlation coefficients (inter-rater reliability, two-way random effect model), *LOA* limit of agreements

## Discussion

Detecting refractive parameters, such as AL and CR, quickly and accurately in children with ametropia is critical. This pilot study is the first to assess the accuracy of Myopia Master in evaluating axial length, keratometry, and refractive measurement in children.

In this study, the AL measured with Myopia Master showed a significant correlation and near-perfect consistency with the other two instruments. Previously, IOLMaster 500 was considered the standard method for AL measurements, adopting the principle of partial coherence interferometry (PCI), with its high precision due to ultrasonic biometric measurement [6, 7]. An infrared light with a wavelength of 760 μm is the light source to measure the optical path length from the anterior surface of the cornea to the retinal pigment epithelium and obtain the AL readings [8, 9]. Based on the principle of swept-source optical coherence tomography (SS-OCT), IOLMaster 700 uses a laser with a wavelength and bandwidth of 1050 nm and 20 nm, respectively, to scan the optical cross-section images at different depths, visualizing the longitudinal sections of the eye structures [10]. Therefore, a higher acquisition rate and a reduced risk of inaccurate measurement due to incorrect fixation can be obtained with this method by imaging the fovea of the macula [10, 11]. PCI is also used in Myopia Master with an 880 nm wavelength. The high consistency between IOLMaster 700 and IOLMaster 500 has been confirmed by previous studies [12, 13]. The mean differences between the AL measured with the three systems were minimal, with no significant difference or clinical significance, and the corresponding deviation of diopter could be ignored [13]. Therefore, the Myopia Master can accurately measure the AL in ametropic children.

The CR measured with Myopia Master also showed a significant correlation and near-perfect consistency with the other two instruments. The CR results obtained with Myopia Master showed no statistically significant or clinical difference from the other two instruments. IOLMaster 700 measures the CR in 18 points in three hexagons (1.5 mm, 2.4 mm, and 3.2 mm) from the center [14]. The mire ring is used in ARK-1 to measure the CR, calculating the mean-weighted power of points on the eight rings 3 mm from the center [15]. Previous studies have proven that the CR readings using IOLMaster 700 could be used as standard data and regarded as a control, as in the current study [16]. The difference between the Myopia Master and the other two devices could be partially explained by the large fluctuation of the K value measured with PCI. Shammas and Chan evaluated the keratometry measured with a PCI device in 121 eyes and reported a 95% LoA range between − 0.55 and + 0.52 D, suggesting that the precision needs to be improved in particularly steep corneas [6]. According to these research findings, we suppose that the Myopia Master could accurately measure the CR in most typical conditions. However, in special cases, such as eyes with steep corneas, adjustments could be needed for CR evaluations.

There was a significant difference in SE measurements between Myopia Master and ARK-1 or manifest refraction; significant differences were also noticed in cylinder measurements (Myopia Master vs. manifest refraction and ARK-1 vs. manifest refraction). No statistically significant differences were found in the spherical and SE measurements between ARK-1 and manifest refraction. The results of our study are consistent with previous findings [4]. As the consistency between Myopia Master and manifest refraction is adequate, monitoring parameters with Myopia Master could help acquire more accurate results in clinical practice. Thus, the correlation and consistency between the three systems showed they can be interchangeable, and the SE measured with the Myopia Master is also feasible for clinical application.

There were some limitations in the present study. First, the sample size was relatively small; further studies using larger databases are warranted to provide more information and detailed results at different ages. Second, the selected population was children with ametropia, and adults were not included for comparative analysis. The consistency between the conventional measurements and Myopia Master in adults needs to be verified.

In conclusion, Myopia Master, as an integrated three-in-one system for AL, CR and SE measurement, can provide multiple biometrical parameters in a single assessment with high efficiency and accuracy.

## Data Availability

The datasets generated and/or analysed during the current study are not publicly available due funding requirement but are available from the corresponding author on reasonable request.

## References

[1] Resnikoff S (2008). Global magnitude of visual impairment caused by uncorrected refractive errors in 2004. B World Health Organ.

[2] Gwiazda J, Hyman L, Hussein M, Everett D, Norton TT, Kurtz D, Leske MC, Manny R, Marsh-Tootle W, Scheiman M (2003). A randomized clinical trial of progressive addition lenses versus single vision lenses on the progression of myopia in children. Invest Ophthalmol Vis Sci.

[3] Lam AK, Chan R, Pang PC (2001). The repeatability and accuracy of axial length and anterior chamber depth measurements from the IOLMaster. Ophthalmic Physiol Opt.

[4] Paudel N, Adhikari S, Thakur A, Shrestha B, Loughman J (2019). Clinical Accuracy of the Nidek ARK-1 Autorefractor. Optometry Vision Sci.

[5] McGraw KO, Wong SP (1996). Forming inferences about some intraclass correlation coefficients. Psychol Methods.

[6] Shammas JH, Chan S (2010). Precision of biometry, keratometry, and refractive measurements with a partial coherence interferometry–keratometry device. J Cataract Refr Surg.

[7] Vogel ADHKF (2001). Reproducibility of optical biometry using partial coherence interferometry: intraobserver and interobserver reliability. J Cataract Refract Surg.

[8] Santodomingo-Rubido J, Mallen EAH, Gilmartin B, Wolffsohn JS (2002). A new non-contact optical device for ocular biometry. Brit J Ophthalmol.

[9] Haigis W, Lege B, Miller N, Schneider B (2000). Comparison of immersion ultrasound biometry and partial coherence interferometry for intraocular lens calculation according to Haigis. Graefes Arch Clin Exp Ophthalmol.

[10] Akman A, Asena L, Güngör SG (2016). Evaluation and comparison of the new swept source OCT-based IOLMaster 700 with the IOLMaster 500. Brit J Ophthalmol.

[11] Kurian M, Negalur N, Das S, Puttaiah NK, Haria D, J TS, Thakkar MM. Biometry with a new swept-source optical coherence tomography biometer: Repeatability and agreement with an optical low-coherence reflectometry device. J Cataract Refract Surg. 2016;42(4):577-81. 10.1016/j.jcrs.2016.01.038 27113881

[12] Jeon HS, Hyon JY, Yoon DY (2020). Comparison of Ocular Biometry and Refractive Outcomes Using IOL Master 500, IOL Master 700, and Lenstar LS900. Korean J Ophthalmol.

[13] Lee AC, Qazi MA, Pepose JS. Biometry and intraocular lens power calculation. Curr Opin Ophthalmol. 2008;19(1)13–17. 10.1097/ICU.0b013e3282f1c5ad 18090891

[14] Hoffer KJ, Hoffmann PC, Savini G (2016). Comparison of a new optical biometer using swept-source optical coherence tomography and a biometer using optical low-coherence reflectometry. J Cataract Refr Surg.

[15] Oshika T, Tomidokoro A, Maruo K, Tokunaga T, Miyata N (1998). Quantitative evaluation of irregular astigmatism by fourier series harmonic analysis of videokeratography data. Invest Ophth Vis Sci.

[16] Dehnavi Z, Khabazkhoob M, Mirzajani A, Jabbarvand M, Yekta A, Jafarzadehpur E (2015). Comparison of the Corneal Power Measurements with the TMS4-Topographer, Pentacam HR, IOL Master, and Javal Keratometer. Middle East Afr J Ophthalmol.

